# Melanoma Diagnosis Using Deep Learning and Fuzzy Logic

**DOI:** 10.3390/diagnostics10080577

**Published:** 2020-08-09

**Authors:** Shubhendu Banerjee, Sumit Kumar Singh, Avishek Chakraborty, Atanu Das, Rajib Bag

**Affiliations:** 1 Department of CSE, Narula Institute of Technology, Kolkata 700109, India; mitsusingh19@gmail.com; 2 Department of Basic Science and Humanities, Narula Institute of Technology, Kolkata 700109, India; avishek.chakraborty@nit.ac.in; 3 Department of MCA, Netaji Subhash Engineering College, Kolkata 700152, India; atanudas75@gmail.com; 4 Department of CSE, Supreme Knowledge Foundation Group of Institutions, Mankundu 712139, India; rajib.bag@gmail.com

**Keywords:** skin cancer, melanoma, skin lesion segmentation, YOLO, deep learning

## Abstract

Melanoma or malignant melanoma is a type of skin cancer that develops when melanocyte cells, damaged by excessive exposure to harmful UV radiations, start to grow out of control. Though less common than some other kinds of skin cancers, it is more dangerous because it rapidly metastasizes if not diagnosed and treated at an early stage. The distinction between benign and melanocytic lesions could at times be perplexing, but the manifestations of the disease could fairly be distinguished by a skilled study of its histopathological and clinical features. In recent years, deep convolutional neural networks (DCNNs) have succeeded in achieving more encouraging results yet faster and computationally effective systems for detection of the fatal disease are the need of the hour. This paper presents a deep learning-based ‘You Only Look Once (YOLO)’ algorithm, which is based on the application of DCNNs to detect melanoma from dermoscopic and digital images and offer faster and more precise output as compared to conventional CNNs. In terms with the location of the identified object in the cell, this network predicts the bounding box of the detected object and the class confidence score. The highlight of the paper, however, lies in its infusion of certain resourceful concepts like two phase segmentation done by a combination of the graph theory using minimal spanning tree concept and L-type fuzzy number based approximations and mathematical extraction of the actual affected area of the lesion region during feature extraction process. Experimented on a total of 20250 images from three publicly accessible datasets—PH2, International Symposium on Biomedical Imaging (ISBI) 2017 and The International Skin Imaging Collaboration (ISIC) 2019, encouraging results have been obtained. It achieved a Jac score of 79.84% on ISIC 2019 dataset and 86.99% and 88.64% on ISBI 2017 and PH2 datasets, respectively. Upon comparison of the pre-defined parameters with recent works in this area yielded comparatively superior output in most cases.

## 1. Introduction

Though the past two decades have seen promising possibilities in the treatment effectiveness and patient quality of life, cancer treatment continues to be a challenge for researchers worldwide. The incidence of skin cancer is higher than that of all other cancers combined. According to reports of the World Health Organization (WHO), skin cancer accounts for one third of all types of cancers happening worldwide with its influence only on an increase with time [1].The three most commonly reported skin cancers are basal cell carcinoma (BCC), squamous cell carcinoma (SCC) and malignant melanoma (MM), of which BCC and SCC account for non-melanocytic cancer [2].The vast majority of skin cancers are non-melanocytic. Melanoma, though less common, is the most fatal of skin cancers and is the cause of maximum number of skin cancer deaths. Melanin, produced by melanocytes, is a prominent skin constituent. This pigment is present in varying degrees depending upon a population’s historical exposure to the sun and is the determinant of an individual’s skin, hair and eye color. Eumelanin and pheomelanin are the two key chemical forms in which melanin exists. Eumelanin, being a dark pigment is a more effective photo protective factor as compared to pheomelanin which is a light colored pigment. The level of pheomelanin in light and dark skinned people is almost similar but owing to the abundance of epidermal eumelanin in heavily pigmented people, they are less susceptible to the damage caused by the UV rays from the sun or other tanning devices and as such less at risk of developing skin cancer [3]. Melanoma affects both sexes though male patients tend to have a higher mortality rate. Recent studies imply that not only UV-A, but also UV-B radiations may account for skin cancer [4,5]. In addition to radiations, skin cancer can be attributed to other factors like a history of cancerous genes in the family, hair color of patients, increased number of benign melanocytic nevi and dysplastic nevi. As believed, melanoma progression originates in the skin epidermis during the radial growth phase and invades into the dermis during the vertical growth phase when prognosis of the cancer becomes increasingly poor. Melanoma may initiate from an existing mole that changes its size, shape, color or feel over time or from a newly formed mole which appears to be abnormal, black or ‘ugly’. These moles quickly and widely metastasize from skin tissues to various parts of the body including bones and cerebrum. The five-year survival ratio of melanoma cases which have reached an advanced stage is below 15% even till date, while early diagnosis elevates the survival rate to 95%—a clear indication of the fact that the survival rate is directly proportional to the timely detection and treatment of the disease [6,7]. American Cancer Society’s annual report for 2019 estimates around 96,480 new cases of melanoma and might prove to be fatal for 7230 patients [8].Thereby, it becomes absolutely imperative to detect melanoma at its earliest possible stage which again is a daunting task because of its varied complexities of nature and intense rapidity in spreading as compared to other forms of skin cancer.

The methods for gauging skin growth to suspect a prospective melanoma has evolved over the years. Before the dawn of the 20th century, melanomas were normally identified solely by naked eye based on the mole’s characteristic features as its size, bleeding or ulceration. In anticipation of suspicious lesions, the lesion was subject to an invasive method, biopsy, for further analysis. Early prognosis, however, was a farfetched dream during those years because one had to rely solely on manual observation owing to absence of advanced technological hardware and software imaging tools. As years passed, non-invasive techniques like dermoscopy or epiluminescence microscopy came to the fore that implemented more economical equipment with superior accuracy [9]. It works on the principle of transillumination of the lesion area and the analysis of its subtle features by intense magnification. This technique too has its limitations as the accuracy of melanoma diagnosis is estimated to be only about 75–84% [10]. Identification of skin lesion images calls for efficient feature extraction and classifiers and precise color capture. In recent context, accurate melanoma diagnosis has found its way through molecular dermatopathology which calls for a consultation between a pathologist and a dermatologist. With recent advances in the field of immunopathology, the diagnosis method made a distinguished shift from descriptive morphology to molecular histopathology [11]. Though most dermatopathologists are in agreement on histological diagnosis of melanocytic lesions by subjecting them to conventional microscopic analysis, certain melanocytic neoplasms termed atypical melanocytic proliferations call for expert consultation before they can be classified as benign or malignant. This also necessitates the evaluation of the histopathological characteristics with that of clinical and microscopic data. Improper application of molecular diagnosis for the identification of benignity or malignancy could as well be misleading and prone to loss of its utmost utility [12,13]. Gradually with the progress in technology and impact of machine learning on medical science computer aided diagnostics made its way in increasing the speed and accuracy of diagnosis. Numerous systems and algorithms like seven-point checklist, ABCD (Asymmetry, Border irregularity, Color variation and Diameter) rule and the Menzies method have since been proposed and put into effect which have added dimension to the efficiency of the diagnostic system by overcoming the issues of traditional dermoscopy techniques [14,15,16,17]. Though the Computer Aided Diagnostic systems have now been integrated with smart phones, the early systems operated on desktops or workstations which enabled physicians and researchers to detect cancerous lesions not perceptible to the human eye [18,19].

While the paper relies on conventional techniques of computer aided melanoma detection, its uniqueness lies in the fusion of new dimensions with the largely accepted pre-existing methods of cancer detection. With the growing utility of machine learning in medical science and to address the disputes on skepticism and unpredictability in science and engineering, fuzzy set theory plays an essential role in image segmentation problem. Motivated by this uncertainty theory, we were eager to discern whether we could possibly relate the fuzzy parameters in case of image segmentation whenever we desired the best fitted region. We undertook to find answers to questions like how feasible would it be to modify or cut the actual examined portion from a large image using pixel values? How could we relate matrix representation of a graph with the pixel values of the original image and perform the iteration such that we may extract the maximal affected region? The paper deploys a graph theory-based segmentation method namely minimal weight computational algorithm that can point out the affected area from the total image roughly. This algorithm is fully based on matrix construction and computes the minimal pixel weight one by one for the whole figure. Additionally, we set one threshold value in case of minimal weight logically which can select the cancer affected area roughly from the total image. Again, we introduced L-Function fuzzy number for second iteration for which the image segmentation method becomes more accurate as compared to the first approximation. Here we have taken the L-Function fuzzy number with dynamic threshold value to tackle the ambiguous portion and developed defuzzification method of L-Function fuzzy number for the crispification of fuzzy number. Due to the ramification and vagueness of detached things and doubt of human thinking, Zadeh (1965) portrayed this remarkable concept of fuzzy set theory in 1965, which has been successfully and rigorously applied on different fields of science and engineering. In course of time several researchers developed [20,21,22,23,24,25,26,27] lots of interesting results on uncertainty arena.

Researchers, off late, have expressed immense interest in experimenting with sundry image segmentation processes. However, combination of the graph theory using minimal spanning tree concept and L-type fuzzy number-based approximations is something that has probably been incorporated for the first time in any research work for lesion segmentation. In addition, our focus throughout the work has been to integrate as many distinctive and effective ways to detect melanoma at its earliest possible stage of which one is the derivation of the center point of the segmented area for effective understanding of the lesion’s asymmetric pattern and border irregularity. Another one of its kind of feature that forms a part of this paper is that it has endeavored to mathematically demonstrate the particularly affected region by calculation of the specific lesion area during feature extraction which has been carried out using the conventional ABCD clinical guide of melanoma diagnosis. Lastly is our choice of open source deep learning-based convolutional neural networkYOLOv3 as a classifier whose architecture is more akin to that of a fully convolutional neural network (FCNN) and is capable of subjugating other top detection methods [28].This classifier extensively speeds up the classification process giving minimum room for errors as compared to other CNNs. The integration of these features within the work scope has significantly assisted in expediting the detection process of melanomatic lesions, which is the fundamental objective of the paper. The entire work has been conceptualized in three sections—proposed methodology, result analysis and conclusion—with each section dealing specifically and elaborately on the focused subject.

## 2. Preliminaries

### 2.1. Definition of Interval Number

An interval number X is denoted by $\left[X_{L},X_{R}\right]$ and defined as $X=\left[X_{L},X_{R}\right]=\left\{x:X_{L}\leq x\leq X_{R},x\in R\right\}$, where R is the set of real numbers and $X_{L}$ and $X_{R}$ generally denotes the left and right range of the interval, respectively.

#### Lemma

The interval $\left[X_{L},X_{R}\right]$ can also be represented as $P\left(\alpha \right)={\left(X_{L}\right)}^{1-\alpha }{\left(X_{R}\right)}^{\alpha }$ for α∈[0,1].

### 2.2. Definition of Fuzzy Set

Let $\tilde{A}$ be a set such that $\tilde{A}=\left\{\left(a,\alpha_{\tilde{A}}\left(\beta \right)\right):a\epsilon A,\alpha_{\tilde{A}}\left(\beta \right)\epsilon \left[0,1\right]\right\}$ which is normally denoted by this ordered pair $\left(a,\alpha_{\tilde{A}}\left(\beta \right)\right)$, here a is a member of the set A and $0\leq \alpha_{\tilde{A}}\left(\beta \right)\leq 1$, then set $\tilde{A}$ is called a fuzzy set.

### 2.3. Definition of Fuzzy Number

Let $\tilde{A}\in F\left(R\right)$ be called a fuzzy number where R denotes the set of real numbers if

- $\tilde{A}$ is normal. That is, $x_{0}\in R$ exists such that $\mu_{\tilde{A}}\left(x_{0}\right)=1$.
- $\mathrm{For}\;\mathrm{all}\;\alpha \in \left(0,1\right],{\;A}_{\alpha }\;\mathrm{is}\;a\;\mathrm{closed}\;\mathrm{interval}$.

### 2.4. Definition of Triangular Fuzzy Number

A triangular fuzzy number $\tilde{A}=\left(s_{1},s_{2},s_{3}\right)$ should satisfy the following conditions:

- $\mu_{\tilde{A}}\left(x\right)$ is a continuous function which is in the interval [0,1].
- $\mu_{\tilde{A}}\left(x\right)$ is a strictly increasing and continuous function on the interval $\left[s_{1},s_{2}\right]$.
- $\mu_{\tilde{A}}\left(x\right)$ is a strictly decreasing and continuous function on the interval $\left[s_{2},s_{3}\right]$.

### 2.5. Definition of Linear Triangular Fuzzy Number (TFN)

**Figure 1:**
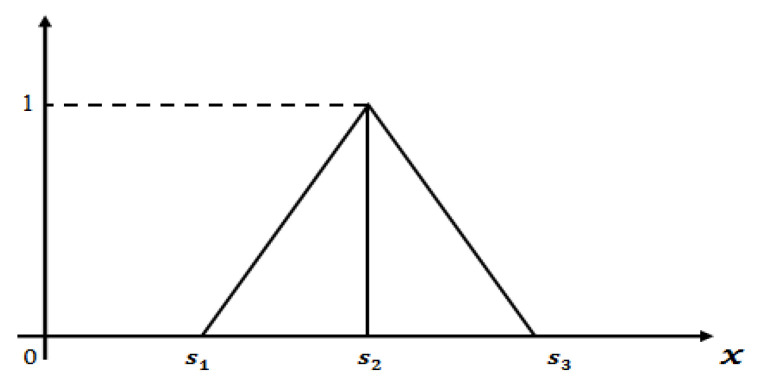
Graphical representation of linear triangular fuzzy number.

A linear triangular fuzzy number (see Figure 1) can be written as ${\tilde{A}}_{\mathrm{TFN}}=\left(s_{1},s_{2},s_{3}\right)$ whose membership function is defined as follows:

(1) $$\mu_{{\tilde{A}}_{\mathrm{TFN}}}\left(x\right)=\{\begin{array}{c} \frac{x-s_{1}}{s_{2}-s_{1}}\mathrm{if}s_{1}\leq x\leq s_{2}\\ 1\;\mathrm{if}x=s_{2}\\ \begin{array}{c} \frac{s_{3}-x}{s_{3}-s_{2}}\mathrm{if}s_{2}\leq x\leq s_{3}\\ 0\;\mathrm{Elsewhere} \end{array} \end{array}$$

### 2.6. Definition of α-cut Form of Linear TFN

α-cut or parametric form of TFN is defined as

(2) $$A_{\alpha }=\left\{x\in X|\mu_{{\tilde{A}}_{\mathrm{TFN}}}\left(x\right)\geq \alpha \right\}=\{\begin{array}{c} A_{L}\left(\alpha \right)=s_{1}+\alpha \left(s_{2}-s_{1}\right)\;\mathrm{for}\;\alpha \in \left[0,1\right]\\ A_{R}\left(\alpha \right)=s_{3}-\alpha \left(s_{3}-s_{2}\right)\;\mathrm{for}\;\alpha \in \left[0,1\right] \end{array}$$

where $A_{L}\left(\alpha \right)$ is the increasing function with respect to α and, $A_{R}\left(\alpha \right)$ is the decreasing function with respect to α.

## 3. Implementation of YOLOv3 Classifier

**Figure 2:**
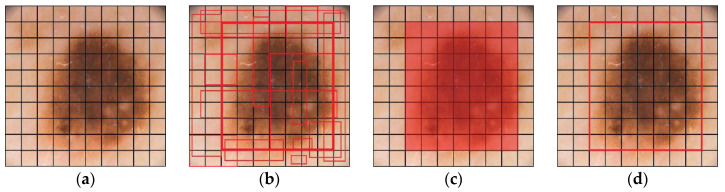
A sample representation (image IMD002) of skin lesion location detection by YOLO. (**a**) S × S grid input, (**b**) bounding boxes are generated with confidence score, (**c**) class probability mapping, (**d**) final lesion location detection.

As mentioned earlier early detection of melanoma plays a vital role in decreasing the mortality rate. Though neural networks like Support Vector Machine, k-nearest neighbor (kNN), decision trees have proved to be efficient classifiers, we in our work have opted for the use of You Only Look Once (YOLO) whose system is organized like a regular CNN, containing convolutional and max-pooling layers and further two completely associated CNN layers. It uses a regression-based algorithm which scans the entire image and makes presumptions to identify, localize and classify objects inside the image (see Figure 2). It is easier to optimize than most classifier algorithms, as it depends on one that utilizes just a single neural network to run sundry components involved in the task. Not only does it yield results at a faster pace (45 frames per second) and have superior accuracy as compared to classification-based algorithms like R-CNN (47 s per individual test image), but it can also be used for real time object detection. Object detection implies determination of positions on the image where certain objects are placed and categorizing of those objects. Here, detection of objects on a particular image is done by YOLOv3 from image pixels to bounding box coordinates and class probabilities, summarizing the detecting process into a single regression problem. The input image is positioned as per S × S grid of cells. For each entity that is available on the image, one grid cell is liable for its prediction. That is where the center of the object falls into.

**Figure 3:**
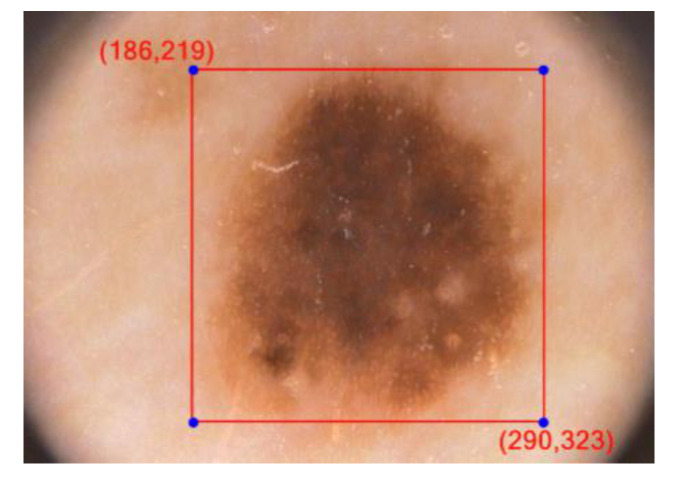
Bounding box in YOLOv3 for image IMD002.

Every framework cell predicts ‘B’ jumping boxes just as ‘C’ class probabilities. The bouncing box forecast has 5 segments: (x, y, w, h, confidence). In this way there are S × S × B × 5 outputs associated with bounding box predictions. The coordinates (x, y) denote the center of the box, relative to the grid cell location, w and h represent the width and height of the bounding box (see Figure 3).

The confidence score refers to the existence or absence of an object within the bounding box. The confidence score can be defined as *Pr*(*Object*) × *IOU*(*pred*, *truth*). In case of absence of any object within the cell, the confidence score should be zero. In other cases, it would be equivalent to the intersection over union (IOU) between the ground truth and the predicted box. Computing intersection over union, which is nothing but a ratio, can therefore be determined via: IOU = Overlap Area/Union Area.

Here, in the numerator, overlapping region between the anticipated bounding box and the ground-truth bounding box is calculated and the denominator denotes the union area, which is the area comprising of both the ground-truth bounding box and the predicted bounding box. Division of the overlap area by the union area is the resultant final score—the intersection over union (IOU).

It is additionally important to anticipate the class probabilities, *Pr*(*Class*(*i*) | *Object*). If no entity is available on the grid cell, the loss function will not penalize it for an off-base class prediction. The network functions by predicting only one set of probabilities in each cell irrespective of the count of boxes B. That creates S × S × C class probabilities. Adding the class predictions to the resultant vector, we get an S × S × (B × 5 + C) tensor as output.

## 4. Proposed Methodology

### 4.1. Training YOLOv3 with PH2, ISBI 2017 and ISIC 2019 Dataset

Skin cancer detection having emerged as a poignant area of research in medical imaging, training the system with the appropriate datasets of relevant images has always proved to be a perplexing task. The classifier was trained with a holdout dataset and the research was conducted with a total of 20,250 images of melanoma and non-melanomatic lesions available from the three publicly accessible holdout datasets—PH2, ISBI 2017 and ISIC 2019. The testing data of melanoma and non-melanomatic images alone accounts for 2530 images. The dataset PH2 (Table 1) comes with a total of 200 images, comprises of 80 atypical nevi, 80 normal nevi and 40 instances of melanoma. The ISBI 2017 (Table 2) comprises of 2750 images where 2000 images are for training, 600 for testing and 150 for validation. The ISIC 2019 dataset originally consists of a total of 25,331 images (Table 3), which is broadly classified into categories of 4522 melanomatic and 20,809 non-melanomatic images. Since we already had 1626 non-melanomatic images to work on from ISBI 2017 dataset and a mere 374 images of melanoma, we restricted our selection of images (Table 4) in ISIC 2019 dataset to all the available 4522 melanoma images in the dataset and randomly chosen 12,778 non-melanomatic images which brought our tally to 17,300 images. Owing to the limited selection of images in each case of melanomatic and non-melanomatic lesions, we categorized our selection of each section (melanoma’s 4522 images and non-melanoma’s 12,778 images) as 80% for training, 10% for testing and another 10% for validation (in approximation). The classifier, thereby, was trained with 13,840 training images, 1730 testing and another 1730 validation images from the ISIC 2019 dataset. Table 5 projects the proposed work’s distribution of selected melanomatic and non-melanomatic images taken from the three datasets for training, validation and testing. These dermoscopic images of 24-bit RGB come with a resolution ranging between 540 × 722 and 4499 × 6748.

All images belonging to these three datasets with varied resolutions were first resized to 512 × 512 resolutions before making them undergo training with YOLOv3.After conversion, YOLOv3 was trained with the resized dataset images. YOLOv3 has been trained on the following parameters: batch size = 64, subdivisions = 16, momentum = 0.9, decay = 0.0005, learning rate = 0.001. YOLOv3 was trained through 70,000 epochs. Based on the results, a conclusion was drawn that the weights saved at the 10,000th epoch proved to be the most efficient detector of location of a lesion within the image.

### 4.2. Pre-Processing

**Figure 4:**
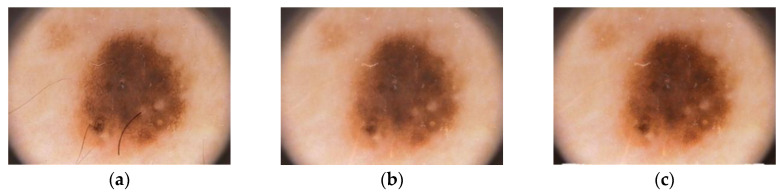
Skin lesion pre-processing method. (**a**) Input image IMD002, (**b**) hair removal by DullRazor algorithm, (**c**) enhanced image.

Since diagnosis of skin cancer with the naked eye can be perplexing, medical professionals often resort to dermoscopy, which nonetheless, is an expensive option. Recent researches have made way for economical substitutes of dermatoscopy, without compromising on the image quality. Here, we employ the ‘tape dermatoscopy’ method introduced by Blum [29] for recording images. The simple yet effective method uses a transparent adhesive over the suspected lesion after application of an immersion fluid over the region. The camera is then placed at an angle of about 45° maintaining a distance of 75 to 85 mm from the surface of the affected skin. Ensuring adequate presence of light, the images of regions bearing suspicious cancerous lesions are then captured for analysis. For quality output, it is advisable to capture the images without zooming in. We used camera with 18mm DX lens, shutter speed of 1/30 ISO-900 and a focal length of 3.5.Upon capturing the image, the focal length and distance of the object from the camera is preserved for further calculations. The main intent behind pre-processing of the captured images is the elimination of noise, undesired artefacts and image augmentation by adjusting the contrast. Here, we have resorted to three significant steps for pre-processing of the derived image. In the first step, we use DullRazor algorithm for removal of hair from over the lesion area. This algorithm first identifies the hair locations with the assistance of a grey morphological closing operation and then verifies the same by distinguishing the identified pixels based on the length and thickness of the detected shape. These pixels are then replaced using bilinear interpolation method and then smoothened with an adaptive median filter. In the next step, image augmentation is performed through histogram equalization. The final stage of image pre-processing involves lesion area detection with the use of YOLOv3′s exclusive feature IOU. The above chronological outputs have been elaborated here under (see Figure 4).

### 4.3. Segmentation

After complete pre-processing of the image, the boundary of the affected area is identified by the process of segmentation. Image segmentation is done for dissection of the primary affected area with high correlation and Region of Interest (RoI). The conventional state-of-art skin lesion segmentation methods like thresholding, region enhancing and clustering did not quite succeed in resolving the complex issues concerning melanoma detection and fell apart mainly owing to their time and computational complexity. As time progressed, these conventional methods were gradually taken over by several well-known methods namely automated computer aided method, k-mean algorithm, convolution, saliency and deconvolution networks [30,31,32,33,34,35,36] and segmentation algorithms like edge detection, thresholding and active contour methods. In recent times, active contour algorithms based on parametric or geometric curve tracking methods have gained immense popularity notwithstanding its mathematical complexity in solving partial differential equations for curve evolution [37,38,39,40,41,42,43,44,45].

**Figure 5:**
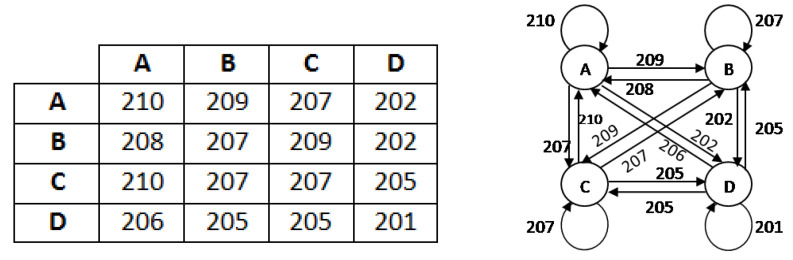
Example of a 4 × 4 sub matrix and its graphical representation.

In this work, we seek to put forth a graph-based segmentation algorithm to detect the boundary values of the affected area. For low computational burden, here we select 4 × 4 order sub matrices of the pre-processed image and create a graph of the adjacency matrix using one graph rule as implied figuratively (see Figure 5).

#### 4.3.1. Iteration-I

This phase involves a graph-based model for deriving minimal weight based on threshold value for detection of affected area. To find the minimal weight of the graph we follow the below algorithm:

1. Construct an adjacent matrix.
2. Discard all self loops from the graph and take one minimum edge in place of multi edge expressions.
3. Find one minimum weight from the 1st row and place one connection. In case of a tie, take any one connection arbitrarily.
4. Find one minimum weight from 1st and previously selected vertex row and add it into one. In case of a tie take any one connection arbitrarily, ensuring that it will not form any circuit.
5. Continue this process until all the vertices are covered but does not form any circuit such that it will generate a spanning tree.
6. Then calculate weight

(3) $$W=(\sum_{j=1}^{3}e_{j})$$

which is the minimum weight. 

After computing W of a sub matrix, we consider threshold T and check the inequality W≤T (for all sub matrixes). If the inequality holds, we select the corresponding matrix that will be one desired affected zone of the total image. Thus, we generate the segmented area of any image using this method.

#### 4.3.2. Iteration-II

**Figure 6:**
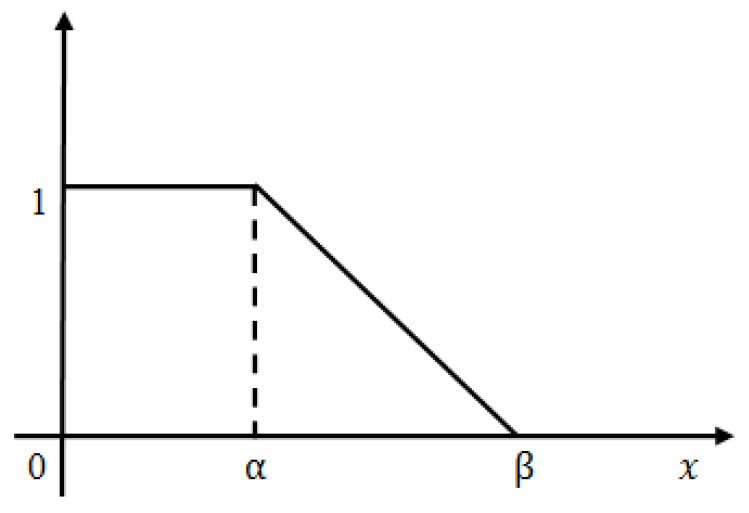
Graphical representation of L-Function fuzzy number.

After finding the affected zone roughly we proceed to further accurate the image segmentation. The iteration-I threshold value is selected hypothetically and is observed that certain non-affected zones are still included within the segmented part. To reduce it we set another threshold value less than T which indicates the fully affected zone. Still the question arises, how much affected zone lies in between iteration-I and iteration-II threshold value? Dilemma remains as to what should be the actual threshold value such that we can take maximum affected zone and discard the maximum non-affected area. In order to overcome this apprehension, we introduced the concept of L-Function fuzzy number (see Figure 6) here to tackle the uncertainty and also developed a de-fuzzification method of L-Function fuzzy number for crispification. This de-fuzzified result actually indicates the threshold value of iteration-II.

- A fuzzy number $\tilde{A}$ is said to be an L-R type fuzzy number if and only if
  (4) $$\mu_{\tilde{A}}\left(x\right)=\{\begin{array}{c} L\frac{\left(m-x\right)}{\alpha },\;\mathrm{for}\;x\leq m,\;\alpha >0\\ R\frac{\left(x-m\right)}{\beta },\mathrm{for}\;x\geq m,\;\beta >0 \end{array}$$
  where, L is for left and R for right reference. M is the mean value of $\tilde{A}$. α,β are called left and right spreads, respectively.
- A fuzzy number $\tilde{A}$ is said to be an L-type fuzzy number if and only if
  (5) $$L\left(x;\alpha ,\beta \right)=\{\begin{array}{c} 1\;,\;x>\alpha \\ \frac{\left(x-\alpha \right)}{\beta -\alpha },\;\alpha \leq x<\beta \\ 0,\;x\geq \beta \end{array}$$

**Figure 7:**
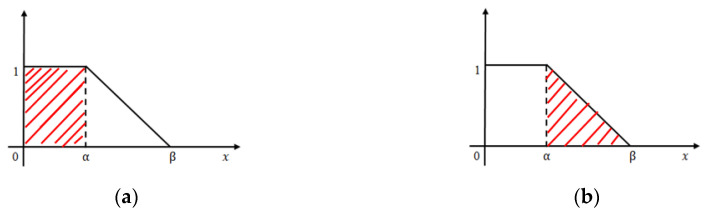
(**a**) Left zone. (**b**) Right zone.

**Figure 8:**
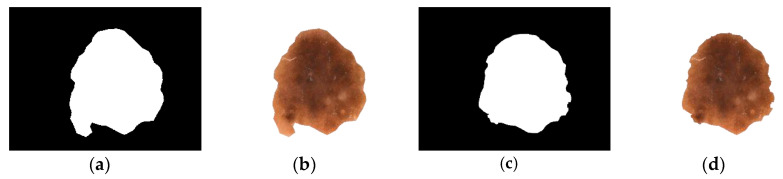
Sequential output of the two phase iteration segmented process. (**a**) Segmented lesion area of iteration I, (**b**) segmented output of iteration I, (**c**) segmented lesion area of iteration II, (**d**) final output of iteration II.

In case of iteration II, we consider the pixel weights of all the sub matrices of the segmented figure. Now, we have a few finite pixel weights and then we select the median weight among all the weights to consider the maximum weight. Next we set the maximum weight in place of β and put the median weight in place of α in this L-type fuzzy number figure. We then proceed to use the de-fuzzification result of the proposed L-type fuzzy number to evaluate the dynamic threshold value of the pixel of the image segmentation computation (see Figure 7). This iteration enables us to select the actual affected zone in a prominent way. L-Function fuzzy number-based segmentation method for second iteration will fetch us more prominent result than the first iteration (see Figure 8).

De-fuzzification of L-typed fuzzy number (area approximation technique): A linear L-type fuzzy number ${\tilde{A}}_{\mathrm{FN}}$ can be converted into a crisp number using the area approximation method. The mathematical formulation is,

(6) $$D=A_{L}\left(\alpha \right)+A_{R}\left(\alpha \right)$$

where,

$$A_{L}\left(\alpha \right)=\mathrm{Area}\;\mathrm{of}\;\mathrm{left}\;\mathrm{Zone}\left(\mathrm{Rectangular}\;\mathrm{shape}\;\mathrm{according}\;\mathrm{to}\;\mathrm{Figure}\;7a\right)=1.\alpha =\alpha$$

$$A_{R}\left(\alpha \right)=\mathrm{Area}\;\mathrm{of}\;\mathrm{Right}\;\mathrm{Zone}\left(\mathrm{Triangular}\;\mathrm{area}\;\mathrm{according}\;\mathrm{to}\;\mathrm{Figure}\;7b\right)=\frac{1}{2}\left(\beta -\alpha \right).1=\frac{\left(\beta -\alpha \right)}{2}$$

Thus,

(7) $$Defuzzificationvalue\;D=A_{L}\left(\alpha \right)+A_{R}\left(\alpha \right)=\frac{\left(\beta +\alpha \right)}{2}$$

### 4.4. Feature Extraction

Since early detection of lesion is a crucial step in the field of skin cancer treatment, right feature extraction can be a vital tool for exploration and analysis of the image. Dermoscopy plays a vital role in examination and inspection of superficial skin lesions significantly improving the sensitivity and specificity of experts for diagnosis of melanoma. A widely accepted rule for feature extraction is the ABCD rule of clinical diagnosis [46,47]. It defines the basis for diagnosis of disease and is a rather safe method as it can be easily detected visually without performing any penetration in the body. This rule fittingly addresses the fundamental question in dermoscopy of whether a melanocytic skin lesion is benign, suspicious (borderline) or malignant. The rule was first introduced in 1985 as the ABCD rule by Stolz and then expanded in 2004 to the ABCDE rule, encompassing several clinical features of melanoma, including Asymmetry, Border irregularity, Color variation, Diameter greater than 6mm and Evolving (a new or changing lesion) [48,49,50]. In its initial stage, detection of melanoma is challenging owing to its small size and symmetry in shape and color. Though the dermoscopic features of melanoma vary widely, the major features of melanoma may include blue-white veil, irregular dots or blotches, atypical pigment network, regression or crystalline structures. As the tumor progresses with time, it begins to acquire more visible dermoscopic features like asymmetry in lesion shape and structure, presence of more than two colors that could be analyzed by ABCD rule [51,52,53]. Apart from the ABCDE rule, other recognized methods and algorithms like Pattern analysis, CASH (Color, Architecture, Symmetry, and Homogeneity) Algorithm, Glasgow seven point checklist, Menzies’ method have also been in vogue from time to time, of which pattern analysis is as old and as widely adopted as the ABCD rule. While the CASH set of laws recognizes the Color, Architectural disorder, Symmetry and Homogeneity/Heterogeneity mole formations, the Glasgow seven-factor checklist implements analysis on three key features (trade in length of lesion, irregular pigmentation and abnormal border) and four minor capabilities (inflammation, itching sensation, diameter greater than 7 mm and discharge of lesions) [54,55,56]. The mole categorization is done based on pattern, symmetry and one color by Menzies technique. However, owing to the complexities of these methods and simplicity of implementation of the ABCD rule, the latter is the most acknowledged among all computerized methods for ruling out melanoma. 

Considering the above dermoscopic features of melanomatic cells, for its clinical diagnosis we resort to the ABCD method of feature extraction post segmentation. In the next step we match the derived segmented area to ensure whether it satisfies the parameters of a melanomatic lesion. Additionally, we have attempted to extract the area of the actual affected region for precise detection of the lesion.

#### 4.4.1. Asymmetry and Border

**Figure 9:**
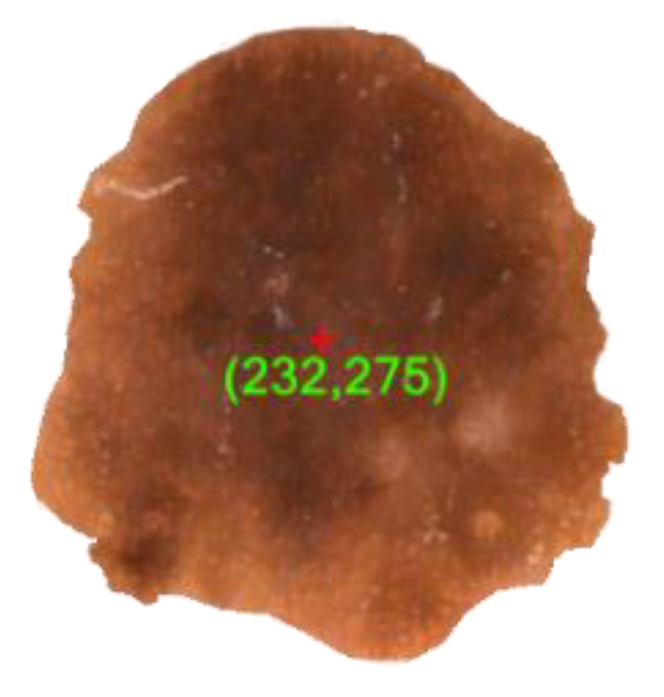
Center point detection of a segmented lesion.

Most melanomas, unlike a round to oval symmetrical common mole, are asymmetrical. If one somehow managed to draw a line through the center of the lesion, the two parts will not match. In addition, melanomic borders tend to be rough and may have notched or jagged edges while basic moles have even boundaries. For detection of the asymmetric shape and border irregularity of the lesion, we have first calculated the center coordinate $\left(x_{0},y_{0}\right)$ of the segmented area (see Figure 9). Next we draw multiple straight lines at any angle between 0° to 180° through the center coordinate which invariably dissects the boundary of the lesion at least two points $\left(x_{k1},y_{k1}\right)\&\left(x_{k2},y_{k2}\right)$. Let the distance of $\left(x_{k1},y_{k1}\right)\&\left(x_{k2},y_{k2}\right)$ from $\left(x_{0},y_{0}\right)$ be $d_{k1}\&d_{k2}$, respectively. Now if in case $d_{k1}\neq d_{k2}$, in maximum cases we can safely deduce that the shape of the lesion is asymmetrical and border is irregular. The mathematical illustrations for center point calculation, asymmetry and border detection are elaborated as follows:

Here, we proposed a new method for center calculation of the examined image. To compute the center of the segmented image we consider the co-ordinates of all points within the segmented portion. Let us assume that, $\left(x_{1},y_{1}),\;(x_{2},y_{2}),(x_{3},y_{3}),\ldots \ldots ,(x_{n},y_{n}\right)$ are the components of the examined image and we want to calculate the center coordinate $\left(x_{0},y_{0}\right)$ using the concept of resultant computational method. Let, $x_{0}$ be the x-coordinate of the center and it will be calculated from the all examined $x_{i}$ components where i∈N and simultaneously $y_{0}$ be the y-coordinate of the center and it will be calculated from the all examined $y_{i}$ components. Computation of $x_{0}$ is given below.

If we consider any two points $\left(x_{1},\;y_{1}\right)\;and\;\left(x_{2},\;y_{2}\right)$ then the coordinate of the resultant will be $\left(\frac{x_{1}+x_{2}}{2},\frac{y_{1}+y_{2}}{2}\right)$. Considering this point and another point $\left(x_{3},\;y_{3}\right)$, if we compute the mid-point then we get the coordinate of the new resultant as $\left(\frac{\frac{x_{1}+x_{2}}{2}+x_{3}}{2},\frac{\frac{y_{1}+y_{2}}{2}+y_{3}}{2}\right)$. Proceeding in this way, the next step is described as follows,

(8) $$\mathrm{For}\;x_{1},x_{2},x_{3}\&x_{4}=\frac{x_{1}+x_{2}+2x_{3}+4x_{4}}{8}\;\&\;y_{1},y_{2},y_{3}\&y_{4}=\frac{y_{1}+y_{2}+2y_{3}+4y_{4}}{8}$$

(9) $$\mathrm{For}\;x_{1},x_{2},x_{3},x_{4}\&x_{5}=\frac{x_{1}+x_{2}+2x_{3}+4x_{4}+8x_{5}}{16}=\frac{x_{1}+2^{0}x_{2}+2^{1}x_{3}+2^{2}x_{4}+2^{3}x_{5}}{2^{4}}$$

(10) $$\mathrm{and}\;y_{1},y_{2},y_{3},y_{4}\&y_{5}=\frac{y_{1}+y_{2}+2y_{3}+4y_{4}+8y_{5}}{16}=\frac{y_{1}+2^{0}y_{2}+2^{1}y_{3}+2^{2}y_{4}+2^{3}y_{5}}{2^{4}}$$

Again,

(11) $$\mathrm{For}\;x_{1},x_{2},x_{3},x_{4},x_{5}\&x_{6}=\frac{x_{1}+2^{0}x_{2}+2^{1}x_{3}+2^{2}x_{4}+2^{3}x_{5}+2^{4}x_{6}}{2^{5}}$$

(12) $$\mathrm{and}\;y_{1},y_{2},y_{3},y_{4},y_{5}\&y_{6}=\frac{y_{1}+2^{0}y_{2}+2^{1}y_{3}+2^{2}y_{4}+2^{3}y_{5}+2^{4}y_{6}}{2^{5}}$$

Continue the above process up to finite n step, we get the final co-ordinate of the resultant as,

(13) $$x_{0}=\frac{x_{1}+2^{0}x_{2}+2^{1}x_{3}+2^{2}x_{4}+2^{3}x_{5}+2^{4}x_{6}+\ldots \ldots +2^{n-2}x_{n}}{2^{n-1}}$$

(14) $$\mathrm{Following}\;\mathrm{in}\;\mathrm{this}\;\mathrm{way}\;y_{0}=\frac{y_{1}+2^{0}y_{2}+2^{1}y_{3}+2^{2}y_{4}+2^{3}y_{5}+\ldots \ldots +2^{n-2}y_{n}}{2^{n-1}}$$

Thus, we get the center coordinate $x_{0},$$y_{0}$ as,

(15) $$\left(\begin{array}{l} \frac{x_{1}+2^{0}x_{2}+2^{1}x_{3}+2^{2}x_{4}+2^{3}x_{5}+2^{4}x_{6}+\ldots \ldots +2^{n-2}x_{n}}{2^{n-1}},\\ \frac{y_{1}+2^{0}y_{2}+2^{1}y_{3}+2^{2}y_{4}+2^{3}y_{5}+2^{4}y_{6}+\ldots \ldots +2^{n-2}y_{n}}{2^{n-1}} \end{array}\right)$$

Here, we examined the asymmetry and border computation using the concept of straight line rotation through a fixed angle. We know that, the equation of any straight line can be written in the form y=mx+c, where m denotes the gradient and c denotes the intercept on y-axis. First, we consider a straight line passes through the center point i.e., $\left(x_{0},y_{0}\right)$, then the equation can be written as $y_{0}=mx_{0}+c$ or $c=y_{0}-mx_{0}$. Again, the equation of the straight line can be written as

(16) $$y=mx+y_{0}-mx_{0}=m(x-x_{0})+y_{0}$$

Now, we calculate m w.r.t. $y=y_{0}$.

Let the line make an angle α with the line $y=y_{0}$, the above equation can be written as $y=\tan\alpha (x-x_{0})+y_{0}$ where $0\leq \alpha \leq 180^{{}^{\circ}}$.

For example; if $\alpha =5^{{}^{\circ}}$ then the equation of the line will be

(17) $$y=0.0874(x-x_{0})+y_{0}$$

Now substituting the boundary points of the segmented area on the above equation one by one, we will get at least two points in which it will satisfy the equation of the straight line. Let the coordinate of the points be expressed as $\left(x_{ki},y_{ki}\right)|i=1,2,3,\ldots N$.

Suppose $\left(x_{k1},y_{k1}\right)\&\left(x_{k2},y_{k2}\right)$ are the two solution points of a line then we calculate the distance $d_{k1}\&d_{k2}$, respectively from the center point $\left(x_{0},y_{0}\right)$.

Therefore, the distance will be as follows;

(18) $$d_{k1}=\sqrt{{\left(x_{0}-x_{k1}\right)}^{2}+{\left(y_{0}-y_{k1}\right)}^{2}}$$

(19) $$d_{k2}=\sqrt{{\left(x_{0}-x_{k2}\right)}^{2}+{\left(y_{0}-y_{k2}\right)}^{2}}$$

**Figure 10:**
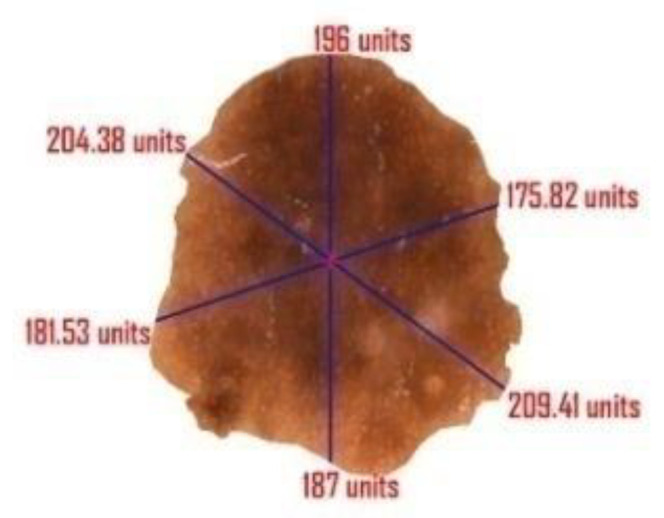
Illustration of multiple lines drawn through the center point of the segmented lesion. All cases of $d_{ki}\neq d_{kj}$ suggest irregular border of the lesion and thereby confirm its asymmetric nature.

After computing the distance, if we observe that $d_{ki}\neq d_{kj}$ in finite cases $k_{i},k_{j}|i,j\in N$, then logically it indicates that the segmented portion is asymmetrical and irregular (see Figure 10).

#### 4.4.2. Color

Multiple colors of a lesion could be a warning sign. While benign moles are generally of a singular brown shade, a melanoma may have various shades of brown, tan or dark. As it grows, the red, white or blue colors may also come into view. In order to match the color of a given lesion with the dataset, our color set includes red, white, dark brown, light brown, black and blue-gray. Sometimes, though, melanomas may lack any pigmentation at all.

To find the multiple colors variations of a lesion we follow the below algorithm.

**Figure 11:**
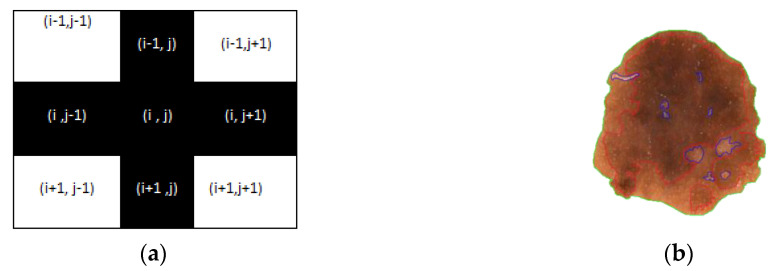
A sample representation of (**a**) plus operation and (**b**) mapping of all color variations within the segmented lesion.

1. Calculate the shape (M × N) of the segmented image X_1_ and every pixel is checked. Simultaneously an image F_1_ (M × N) is generated where $f_{i,j}$ is considered a pixel value at the location (*i*,*j*).
2. Extra border padding in plotting matrix F_1_, for calculation.
3. Calculate RGB value of each pixel $x_{i,j}$ and convert it to corresponding HSV
4. if HSV value of $x_{i,j}$ ranges from 30,62,77 to 30,68,57 then $f_{i,j}$ = 1 // for light brown
5. if HSV value of $x_{i,j}$ ranges from 30,67,51 to 30,67,28 then $f_{i,j}$ = 2 // for dark brown
6. if HSV value of $x_{i,j}$ ranges from 30,67,22 to 30,67,11 then $f_{i,j}$ = 3 // for tan black
7. if HSV value of $x_{i,j}$ ranges from 60,2,17 to 30,0,10 then $f_{i,j}$ = 4 // for blue gray
8. if HSV value of $x_{i,j}$ ranges from 0,100,46 to 0,87,70 then $f_{i,j}$ = 5 // for red
9. if HSV value of $x_{i,j}$ ranges from 0,0,94 to 0,0,98 then $f_{i,j}$ = 6 // for white
10. if $f_{i,j}$ > 0 and lies in the border line then plot that pixel with color according to clusters.
11. else if ($f_{i,j}$ $\leq \frac{f_{i,j-1}+f_{i,j+1}+f_{i-1,j}+f_{i+1,j}}{4}$) then continue // plus (+) operation (see Figure 11a).
12. else plotting the pixel with different colors for different clusters as per Figure 11b.

#### 4.4.3. Diameter

**Figure 12:**
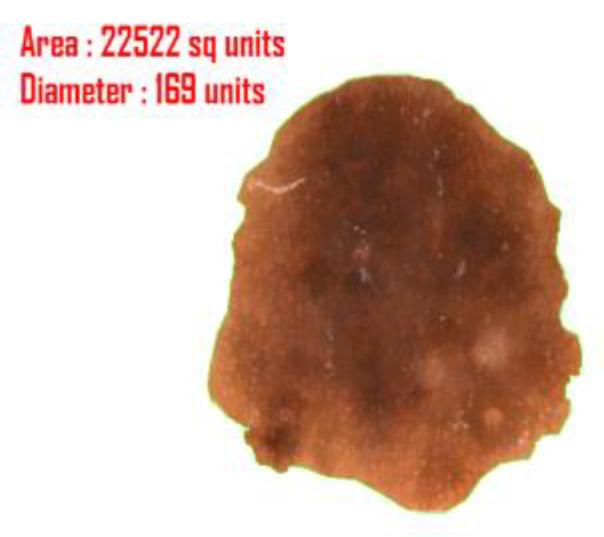
Outcome of diameter and area of the segmented lesion.

Diameter computation is one of the most crucial topics in image segmentation. In case of a suspicious melanomatic lesion, the ‘diameter greater than 6 mm’ feature implies the size of the lesion. For calculation of the diameter of a suspicious lesion, we determine the maximum distance between two pixel values positioned on the border of the lesion and determine the area of the actual affected region. The determination of area is crucial to decipher the actual affected region; since it is not feasible to apprehend the readings of the diameter and the area in pixel value, we rescale the derived figures in terms of millimeter. Here, we compute each distance of the coordinate points $\left(x_{1},y_{1}),\;(x_{2},y_{2}),(x_{3},y_{3}),\ldots \ldots ,(x_{n},y_{n}\right)$ and then compute maximum distance between them. We also incorporated the idea of focal length here to compute the actual length of the segmented image. Further, we will calculate the area of the affected portion using the concept of polyhedron area computation. This noble thought will help the researchers to calculate the extreme distance and actual area of the affected part. The complete derivation of the diameter and the area (see Figure 12) are as follows:

(20) $$d_{1}=\max\left\{\begin{array}{l} \sqrt{{\left(x_{n}-x_{1}\right)}^{2}+{\left(y_{n}-y_{1}\right)}^{2},}\sqrt{{\left(x_{n-1}-x_{1}\right)}^{2}+{\left(y_{n-1}-y_{1}\right)}^{2}}\\ \ldots ,\sqrt{{\left(x_{2}-x_{1}\right)}^{2}+{\left(y_{2}-y_{1}\right)}^{2}},\sqrt{{\left(x_{1}-x_{1}\right)}^{2}+{\left(y_{1}-y_{1}\right)}^{2}} \end{array}\right\}$$

(21) $$d_{2}=\max\left\{\begin{array}{l} \sqrt{{\left(x_{n}-x_{2}\right)}^{2}+{\left(y_{n}-y_{2}\right)}^{2},}\sqrt{{\left(x_{n-1}-x_{2}\right)}^{2}+{\left(y_{n-1}-y_{2}\right)}^{2}}\\ ,\ldots ,\sqrt{{\left(x_{2}-x_{2}\right)}^{2}+{\left(y_{2}-y_{2}\right)}^{2}} \end{array}\right\}$$

Continue the above process until $\frac{n}{2}$ times (if n is even) else $\frac{n+1}{2}$ times.

(22) dn2=max{(xn−xn2)2+(yn−yn2)2,…,(x1−xn2)2+(y1−yn2)2}

Let, d=max{d1,d2,d3,……,dn2} and dr=d|1≤r≤n2.

Find the max $d_{r}$ position. Let $\left(x_{t},y_{t})and(x_{r},y_{r}\right)$ be the extreme points.

The extreme distance is $\sqrt{{\left(x_{t}-x_{r}\right)}^{2}+{\left(y_{t}-y_{r}\right)}^{2}}unit$ where 1≤r≤n2 and 1≤t≤n.

The actual length $L=d\times \frac{f}{u+f}mm$ where u denotes the distance of the object from the camera and f is the focal length of the camera.

Evaluating the total number of coordinates spread over the entire segmented region, we derive the area of the desired portion:

(23) $$\Delta =\frac{1}{2}\left(\left|\begin{array}{cc} x_{1} & x_{2}\\ y_{1} & y_{2} \end{array}\right|+\left|\begin{array}{cc} x_{2} & x_{3}\\ y_{2} & y_{3} \end{array}\right|+\left|\begin{array}{cc} x_{3} & x_{4}\\ y_{3} & y_{4} \end{array}\right|+\left|\begin{array}{cc} x_{4} & x_{5}\\ y_{4} & y_{5} \end{array}\right|+\ldots \ldots +\left|\begin{array}{cc} x_{n} & x_{1}\\ y_{n} & y_{1} \end{array}\right|\right)$$

(24) $$=\frac{1}{2}\left|\sum_{i=1}^{n-1}x_{i}y_{i+1}+x_{n}y_{1}-\sum_{i=1}^{n-1}x_{i+1}y_{i}-x_{1}y_{n}\right|$$

Actual Area $A=\Delta {\left(\frac{f}{u+f}\right)}^{2}mm^{2}$ where u denotes the distance of the object from the camera and f is the focal length of the camera.

In Figure 12, the measurements have been generated in ‘units’ rather than the actual metrics because u and f are unknown.

## 5. Parameters for Performance Evaluation

The methodology adopted for location detection of the lesion by means of YOLOv3 was assessed in two phases. To begin with, the lesion location recognition performance of trained YOLOv3 in skin lesion images, was assessed by inciting IOU metric. The recognized location was asserted if the *IOU* score was more noteworthy than 80%. Secondly, the performance was tested on the predefined parameters to additionally evaluate our technique: sensitivity (*Sen*), specificity (*Spe*), the dice coefficient (*Dic*), the Jaccard index (*Jac*) and accuracy (*Acc*). Here, Sen indicates the measure of accurately segmented lesion, *Spe* is the properly segmented ratio of non-lesion areas, *Dic* is used to quantify segmented lesions and explain ground truth connection and Jac is viewed as an assessment metric for the convergence proportion between the achieved segmentation results and ground truths masks. Finally, accuracy shows the overall pixel-wise segmentation performance. The formula for calculation of the above-mentioned evaluation metrics are as follows.

(25) $$IOU=\frac{\mathrm{Area}\;\mathrm{of}\;\mathrm{Overlap}}{\mathrm{Area}\;\mathrm{of}\;\mathrm{Union}}$$

(26) $$Sen=\frac{TP}{TP+FN}$$

(27) $$Spe=\frac{TN}{TN+FP}$$

(28) $$Dic=\frac{2\times TP}{(2\times TP)+FP+FN}$$

(29) $$Jac=\frac{TP}{TP+FN+FP}$$

(30) $$Acc=\frac{TP+TN}{TP+TN+FN+FP}$$

The *TP*, *TN*, *FP*, *FN* represents true positive, true negative, false positive and false negative, respectively. The lesion pixels in the image are considered as true positive (*TP*) if they are detected/segmented correctly, else they are regarded as false negatives (*FN*). As for non-lesion pixels, in the images they are considered as true negative (*TN*) if they are predicted as non-lesion pixel, else they are regarded as false positive (*FP*).

## 6. Result Analysis

This section rests on the performance analysis of the complete working method projected through this paper and is recorded on the basis of four significant parameters—lesion location detection capacity, segmentation performance, feature extraction accurateness and computational time. Here three different publicly available datasets PH2, ISBI 2017 and ISIC 2019 are used in the detection and segmentation purpose. The whole operations and computations were completed on a PC with i7 processor, 32 GBRAM with 4 GB GPU and Ubuntu 18.04 operating system. The entire system was developed by Python and OpenCv image processing framework.

The recognition execution was determined considering three metrics—sensitivity, specificity and IOU to detect correct lesion in correct order. The PH2 dataset gave a 97.5% sensitivity, 98.5% specificity and 95 IOU in the detection phase. While sensitivity of the proposed system on the ISBI 2017 dataset was 98.47% with specificity of 97.51 % and IOU as 92, the scores in case of ISIC 2019 were 97.77, 97.65 and 90 in order. Table 6 refers to the recognition execution of the model on three datasets.

**Figure 13:**
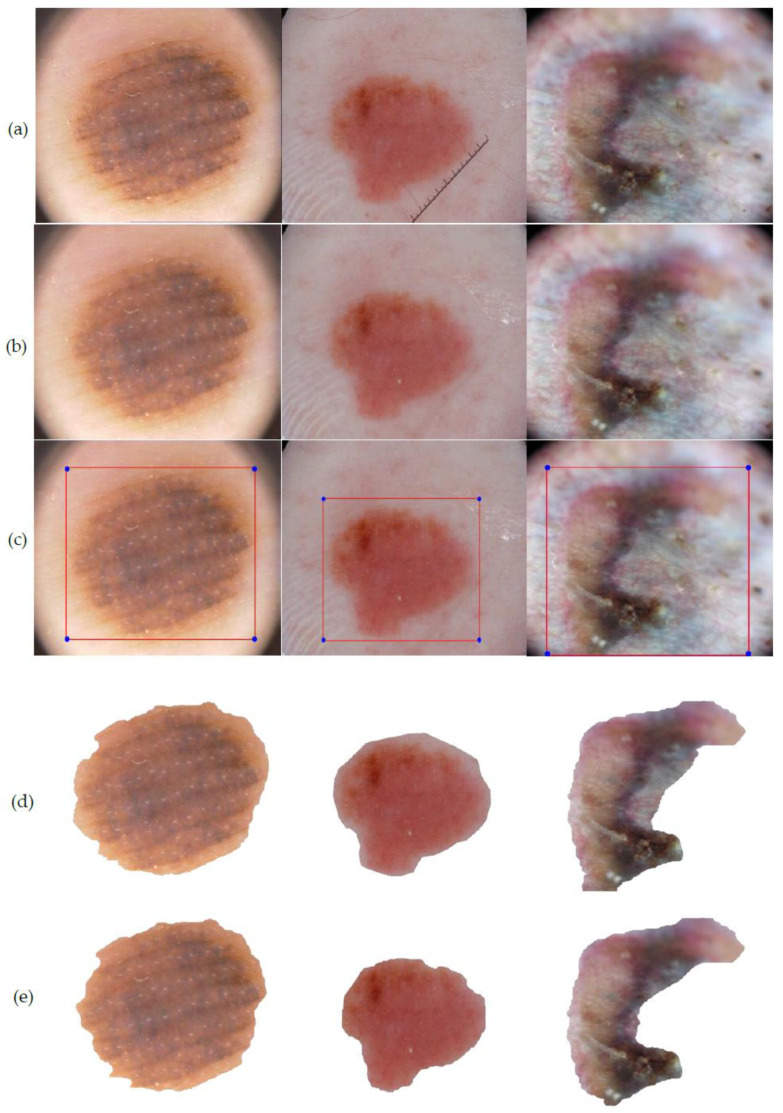
(**a**) Input images (IMD285, ISIC_0013617 and ISIC_0059197), (**b**) output image after hair removal and enhancement, (**c**) location detection by YOLOv3, (**d**) segmented image after iteration I, (**e**) segmented image after iteration II (final segmented output).

**Figure 14:**
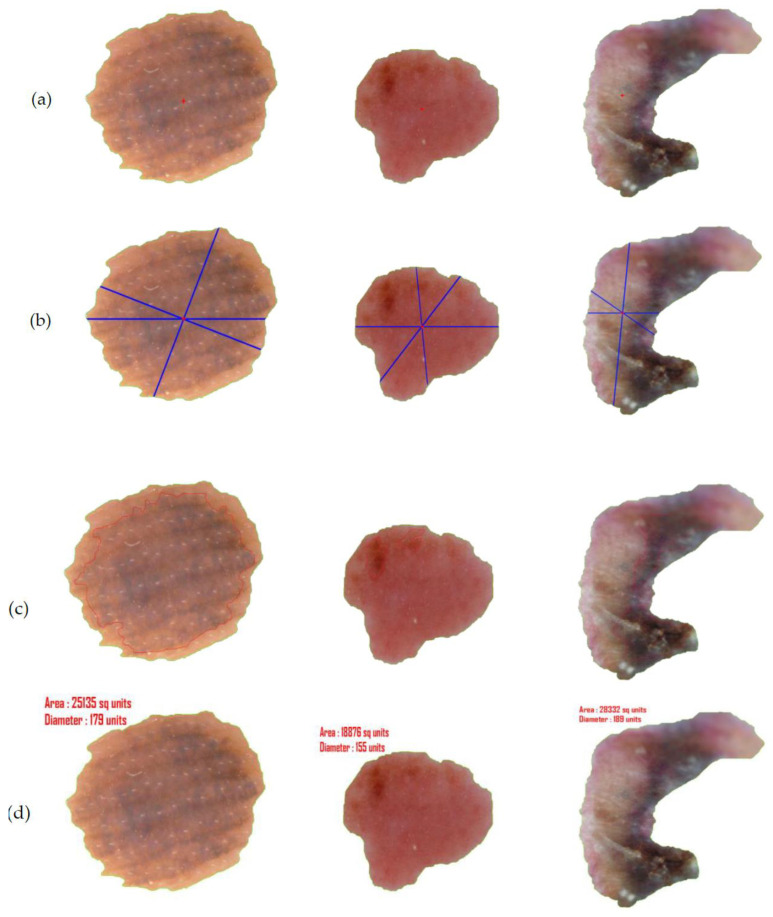
(**a**) Center point detection of the final segmented output, (**b**) asymmetry and border irregularity detection by calculating $d_{ki}$ and $d_{kj}$, (**c**) the color variation detection and segregation on lesion images. (**d**) The derived measurement of diameter and area of the segmented lesion projected in ‘units’.

After assessment of the identification of the lesion location, the segmentation execution of our technique was evaluated on two datasets on the basis of accuracy, sensitivity, specificity, Jac and Dic metrics. Our segmentation method involves two stages—the first being graph-based i.e., iteration I (see Table 7) and the second deals with L-Function fuzzy number in iteration II (see Table 8). The inclusion of the second step is to ensure better segmentation over rest of the methods available in recent times. Table 8 outlines the segmentation performance of the projected pipeline technique. Figure 13 and Figure 14 are illustrative of the instances of the segmentation outputs and feature extraction outcomes of the proposed model.

**Figure 15:**
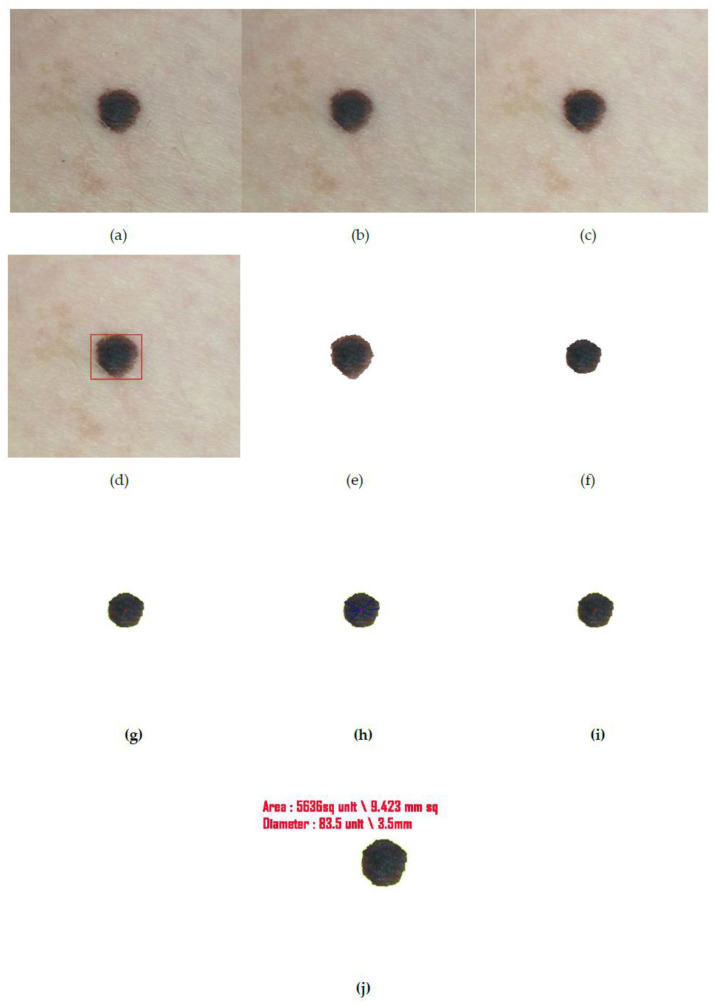
(**a**) Input captured image from camera, (**b**) output image after hair removal, (**c**) enhanced image, (**d**) location detection by YOLOv3, (**e**) segmented image after iteration I, (**f**) segmented image after iteration II (final segmented output), (**g**) center point detection of the final segmented output, (**h**) asymmetry and border irregularity detection by calculating $d_{ki}$ and $d_{kj}$, (**i**) the color variation detection and segregation on lesion images. (**j**) Here the focal length of the camera f and the distance of the object from the camera u is automatically calculated whose values are 3.5 mm and 80 mm, respectively and thereby the diameter and area are also expressed in terms of millimeters.

In addition to conducting the study on images gathered from the datasets, we also have repeated the analysis on image captured in real time in order to overcome the dilemma of producing the measurements in their appropriate forms. As can be observed in the earlier images (see Figure 14), the measurements have merely been projected as ‘unit’. This is because the actual measurement could not be fathomed as focal length of the camera and the distance of the object from the camera could not be calculated from images obtained from datasets. Through Figure 15, however, we are able to project the units of the border and diameter of the real time captured mole authentically which also is a proof of the efficiency of the proposed method.

## 7. Discussion

**Figure 16:**
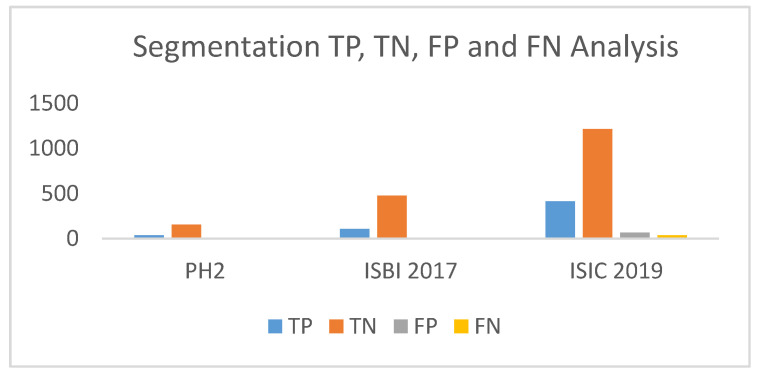
True positive (TP), true negative (TN), false positive (FP), false negative (FN) analysis of the proposed segmentation method.

In recent years, notable contributions have been made by scholars for redefining the segmentation process. Our work was assessed on three well-established publicly available datasets PH2, ISBI 2017 Skin Lesion Challenge (SLC) and ISIC 2019 (SLC). We evaluated our proposed segmentation method against segmentation frameworks based on deep convolutional neural network (DCNN) [57], approaches with U-nets followed by histogram equalization and C-means clustering [58], segmentation done by crowdsourcing from ISIC 2017 challenge results [59], simultaneous segmentation and classification using bootstrapping deep convolutional neural network model [60], segmentation using contrast stretching and mean deviation [61] and semantic segmentation method for automatic segmentation [62]. In addition, we also drew inspiration from few of the most successful lesion segmentation methods introduced in the recent years like segmentation by means of FCN networks, multi stage fully convolution network (FCN) with parallel integration (mFCN-PI) [63,64], FrCN method involving simultaneous segmentation and classification, a fully-convolutional residual networks (FCRN), which was an amendment and extension of FCN architecture [65,66,67], a deep fully convolutional-deconvolutional neural network (CDNN) performing automatic segmentation [68] and lastly with the semi automatic Grab cut algorithm [69]. Table 9 and Table 10 project a comparative study with the aforementioned works based on datasets available from PH2 and ISBI 2017, respectively. Table 11 includes the segmentation performance results of the proposed method on selected images from ISIC 2019. All performances were measured on the pre-defined parameters of accuracy, sensitivity, specificity, Jac and Dic, which in turn were assessed by calculation of TP, TN, FP, FN cases (Figure 16) in instance of each dataset.

As can be perceived from the tabular data, all the above researches accomplished substantially credible results in lesion segmentation by improvising on existing segmentation methods. Comparing the proposed method’s outcome with these contemporary segmentation approaches evidently demonstrates that the former’s performance has an edge over all the existing deep-learning methods available. Adjudging the method’s performance on PH2 dataset, it outperformed the best contributions in sensitivity and specificity scoring 97.5% in each. It also substantially outscored the rest in terms of Jac and Dice score with 88.64% and 93.97% falling behind but only to the inspiring work of Xie who achieved a staggering score of 89.4% and 94.2% in the said parameters. It also achieved second best accuracy with 97.5% behind Hasan’s 98.7%. In addition, the segmentation results evaluated on ISBI 2017 dataset illustrates the proposed method outdoes the rest by a significant margin, including the ones that attained the top three positions in the ISIC 2017 Skin Lesion Challenge, on all parameters with 97.33% accuracy and a Jac score of 86.99%. We attribute the method’s efficiency to the infusion of L-Function fuzzy number in the segmentation method.

Proposed recognition result is compared with different methods of different classifiers like Tree, SVM, KNN and YOLOv3. The different parameters are set to draw comparison between the existing deep-learning models and our proposed method using You Only Look Once (YOLO). Comparison is done on the basis of sensitivity, specificity, precision, accuracy and AUC. Time (in second) is also used as a comparison metric to validate the speed of our method. Table 12, Table 13 and Table 14 draw the comparisons between said classifiers on images belonging to PH2, ISBI 2017 and ISIC 2019 datasets.

**Figure 17:**
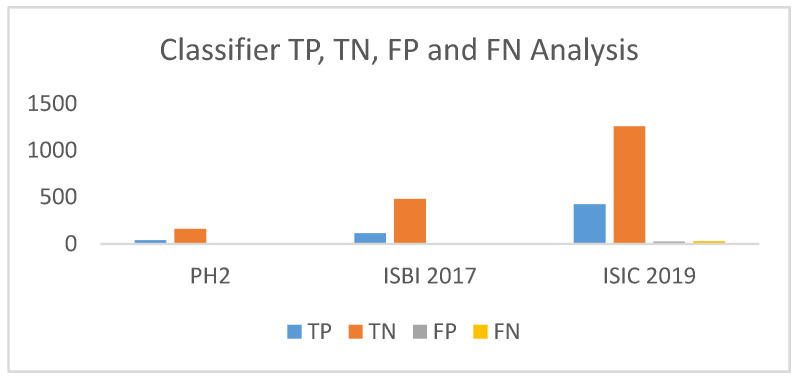
TP, TN, FP, FN analysis of the proposed classifier.

The comparisons clearly depict that the proposed classification method has an edge over all other existing methods of different classifier. Not only does the classifier projects superior output spanning all parameters when contrasted with other efficient classifiers, also the time for detection of melanoma is minimized in case of the proposed method. The analysis of TP, TN, FP and FN derived from the classifier’s performance on the three datasets is projected through Figure 17.

Choosing YOLO as a classifier decreases the detection time and increases the efficiency of skin lesion detection. Use of preprocessing models where automatic hair removal is followed by image enhancement and proper segmentation methods contributed to better accuracy of the proposed method. Proper validation of ABCD features of melanoma by proposed method also add to better result.

## 8. Conclusions

For decades, melanoma incidence has progressively risen and is projected to continue to rise across the world. Melanoma mortality trends are variable and as with incidence, are influenced by geography, ethnicity, age and sex. Attempts to improve the diagnostic accuracy of melanoma diagnosis have spurred the development of innovative ideas to cope up with the fatality of the disease. Research into the causes, prevention and treatment of melanoma is being carried out in medical centers throughout the world. In this article, an efficient mathematical modeling is presented for the purpose of segmentation and feature extraction. The studies have been executed on three distinguished datasets PH2, ISBI 2017 and ISIC 2019.In addition, test results ranging over a multitude of parameters assert that the proposed technique using YOLOv3 accomplished promising outcomes when contrasted with other deep learning-based methodologies. Here, we have examined the computational strides to consequently analyze cancer by utilization of various digital and dermatological images from the aforementioned datasets. The two-phase process combining graph theory and fuzzy number-based approximation heightened segmentation results which in turn positively affect the recognition process classification accuracy. The proposed features in this work have rendered a considerable amount of efficiency to the overall methodology of cancer detection though much remains to be explored, analyzed and accomplished in this area of human health. Future prospects may involve training of the system with wider range of datasets bearing multiple lesions and lesion classification through improved CAD methods or clinical testing.

## Figures and Tables

**Table 1:** Distribution of PH2 dataset.

Datasets	Test Data			Total
Label	B	M	AT	
PH2	80	40	80	200

B—benign, M—melanoma, AT—atypical nevus.

**Table 2:** Distribution of ISBI 2017 dataset.

Datasets	Training Data			Validation Data			Test Data			Total
Label	B	M	SK	B	M	SK	B	M	SK	
ISBI 2017	1372	374	254	78	30	42	393	117	90	2750

B—benign, M—melanoma, SK—seborrheic keratosis.

**Table 3:** Distribution of ISIC 2019 dataset.

Dataset	NV	M	BKL	BCC	SCC	VL	DF	AK	Total
ISIC 2019	12,875	4522	2624	3323	628	253	239	867	25,331

NV—melanocytic nevus, M—melanoma, BKL—benign keratosis, BCC—basal cell carcinoma, SCC—squamous cell carcinoma, VL—vascular lesion, DF—dermatofibroma, AK—actinic keratosis.

**Table 4:** Distribution of selected images from the ISIC 2019 dataset used in the proposed work.

Datasets	Training Data		Validation Data		Test Data		Total
Label	M	NM	M	NM	M	NM	
ISIC 2019	3622	10,218	450	1280	450	1280	17,300

M—melanoma, NM—non-melanoma.

**Table 5:** Distribution of selected images from PH2, ISBI 2017 and ISIC 2019 datasets used in the proposed work.

Datasets	Training Data		Validation Data		Test Data		Total
Label	M	NM	M	NM	M	NM	
PH2	*	*	*	*	40	160	200
ISBI 2017	374	1626	30	120	117	483	2750
ISIC 2019	3622	10,218	450	1280	450	1280	17,300
Total	3996	11,844	480	1400	607	1923	20,250

M—melanoma, NM—non-melanoma, * there are no data in this field.

**Table 6:** Skin lesion location detection performance (%) analysis of YOLOv3.

Datasets	Sensitivity	Specificity	IOU
PH2	97.5	98.75	95
ISBI 2017	98.47	97.51	92
ISIC 2019	97.77	97.65	90

**Table 7:** Segmentation performance (%) of iteration I.

Datasets	Acc	Sen	Spe	Jac	Dic
PH2	96	95	96.25	82.60	90.47
ISBI 2017	95.67	88.89	97.31	80.00	88.89
ISIC 2019	92.94	88.88	94.37	76.62	86.76

**Table 8:** Segmentation performance (%) of iteration II.

Datasets	Acc	Sen	Spe	Jac	Dic
PH2	97.50	97.50	97.50	88.64	93.97
ISBI 2017	97.33	91.45	98.76	86.99	93.04
ISIC 2019	93.98	91.55	94.84	79.84	88.79

**Table 9:** The comparative study of the proposed segmentation performance (%) on PH2 dataset.

References	Year	Dataset	Acc	Sen	Spe	Jac	Dic
Bi et al. (ResNets) [63]	2017	PH2	94.24	94.89	93.98	83.99	90.66
Bi et al. [64]	2019	PH2	95.03	96.23	94.52	85.9	92.1
Saba etal. [57]	2019	PH2	95.41	-	-	-	-
Unver etal. [69]	2019	PH2	92.99	83.63	94.02	79.54	88.13
Xie et al. [60]	2020	PH2	96.5	96.7	94.6	89.4	94.2
Hasan et al. [62]	2020	PH2	98.7	92.9	96.9	-	-
Proposed Method	2020	PH2	97.5	97.5	97.5	88.64	93.97

**Table 10:** The comparative study of the proposed segmentation performance (%) on ISBI 2017 dataset.

References	Year	Dataset	Acc	Sen	Spe	Jac	Dic
Yuan et al. (CDNN) [68] Lin et al. (U-Net) [58] Bi et al.(ResNets) [63] Li et al. [65] Al-Masni et al. [66] Bi et al. [64] Soudani et al. [59] Unver etal. [69] Xie et al. [60] Akram et al. [61] Hasan et al. [62] Al-Masni et al. [67]	2017 2017 2017 2018 2018 2019 2019 2019 2020 2020 2020 2020	ISBI 2017 ISBI 2017 ISBI 2017 ISBI 2017 ISBI 2017 ISBI 2017 ISBI 2017 ISBI 2017 ISBI 2017 ISBI 2017 ISBI 2017 ISBI 2017	93.4 - 93.4 93.2 94.03 94.08 94.95 93.39 94.7 95.9 95.3 81.57	82.5 - 80.2 82 85.4 86.2 85.87 90.82 87.4 - 87.5 75.67	97.5 - 98.5 97.8 96.69 96.17 95.66 92.68 96.8 - 95.5 80.62	76.5 62.00 76 76.2 77.11 77.73 78.92 74.81 80.4 - - -	84.9 77.00 84.4 84.7 87.08 85.66 88.12 84.26 87.8 - - -
Proposed Method	2020	ISBI 2017	97.33	91.45	98.76	86.99	93.04

**Table 11:** The outcome of the proposed segmentation performance (%) on ISIC 2019 dataset.

References	Year	Dataset	Acc	Sen	Spe	Jac	Dic
Proposed Method	2020	ISIC 2019	93.98	91.55	94.84	79.84	88.79

**Table 12:** Comparative study between proposed classifier and well-known classifiers on PH2 dataset.

Classifier	Method	Acc (%)	Sen (%)	Spec (%)	Prec	AUC	Time (in sec)
**TREE**	CT	75	75	75	42.85	0.83	17.11
	ST	76.5	77.5	76.25	44.92	0.89	4.61
**SVM**	LSVM	97	95	97.5	90.47	0.99	7.24
	CSVM	96.5	90	98.12	92.30	0.96	9.43
	QSVM	97.5	95	98.12	92.68	0.98	5.81
	MGSVM	92	90	92.5	75	0.96	5.11
**KNN**	FKNN	98	95	98.75	95	0.97	4.94
	MKNN	96.5	92.5	97.5	90.24	0.98	3.96
	Cosine	96	92.5	96.87	88.09	0.99	4.65
	Cubic	98	95	98.75	95	0.98	5.78
	WKNN	94	97.5	93.12	78	0.99	4.15
**YOLO**	**Proposed Method**	**99**	**97.5**	**99.37**	**97.5**	**0.99**	**2.63**

**Table 13:** Comparative study between proposed classifier and well-known classifiers on ISIB 2017 dataset.

Classifier	Method	Acc (%)	Sen (%)	Spec (%)	Prec	AUC	Time (in sec)
**TREE**	CT	92.83	90.60	93.37	76.81	0.96	8.12
	ST	88.00	88.89	87.78	63.80	0.92	12.67
**SVM**	LSVM	96.67	93.16	97.52	90.08	0.95	11.42
	CSVM	86.83	85.47	87.16	61.73	0.92	140.54
	QSVM	97.50	94.87	98.14	92.50	0.97	21.47
	MGSVM	96.67	94.87	97.10	88.80	0.98	13.45
**KNN**	FKNN	94.00	92.31	94.41	80.00	0.90	9.04
	MKNN	97.83	94.02	98.76	94.83	0.96	10.01
	Cosine	97.50	93.16	98.55	93.97	0.97	10.78
	Cubic	97.50	94.02	98.34	93.22	0.97	102.01
	WKNN	98.50	96.58	98.96	95.76	0.98	12.75
**YOLO**	**Proposed Method**	**99.00**	**97.44**	**99.38**	**97.44**	**0.99**	**7.14**

**Table 14:** Comparative study between proposed classifier and well-known classifiers on ISIC 2019 dataset.

Classifier	Method	Acc (%)	Sen (%)	Spec (%)	Prec	AUC	Time (in sec)
**TREE**	CT	91.68	90.67	92.03	80.00	0.95	15.21
	ST	86.94	89.11	86.17	69.38	0.93	21.88
**SVM**	LSVM	93.47	92.00	93.98	84.32	0.95	19.39
	CSVM	88.09	88.44	87.97	72.10	0.93	246.98
	QSVM	93.06	91.56	93.59	83.40	0.98	37.01
	MGSVM	94.86	91.78	95.94	88.82	0.96	26.32
**KNN**	FKNN	93.82	83.11	97.58	92.35	0.92	18.15
	MKNN	90.69	86.00	92.34	79.79	0.96	17.47
	Cosine	92.02	93.11	91.64	79.66	0.99	19.19
	Cubic	94.74	88.00	97.11	91.45	0.97	176.45
	WKNN	95.84	93.56	96.64	90.73	0.99	22.39
**YOLO**	**Proposed Method**	**97.11**	**94.22**	**98.13**	**94.64**	**0.99**	**12.40**
